# Persistent social isolation reflects identity and social context but not maternal effects or early environment

**DOI:** 10.1038/s41598-017-18104-4

**Published:** 2017-12-19

**Authors:** L. J. N. Brent, A. Ruiz-Lambides, M. L. Platt

**Affiliations:** 1 School of Psychology, Centre for Research in Animal Behaviour, University of Exeter, Exeter, UK; 2 Caribbean Primate Research Center, University of Puerto Rico, San Juan, Puerto Rico; 3 Departments of Neuroscience, Psychology, and Marketing, University of Pennsylvania, Philadelphia, PA USA

## Abstract

Individuals who are well integrated into society have greater access to resources and tend to live longer. Why some individuals are socially isolated and others are not is therefore puzzling from an evolutionary perspective. Answering this question requires establishing the mix of intrinsic and contextual factors that contribute to social isolation. Using social network data spanning up to half of the median adult lifespan in a gregarious primate, we found that some measures of social isolation were modestly repeatable within individuals, consistent with a trait. By contrast, social isolation was not explained by the identity of an animal’s mother or the group into which it was born. Nevertheless, age, sex and social status each played a role, as did kin dynamics and familiarity. Females with fewer close relatives were more isolated, and the more time males spent in a new group the less isolated they became, independent of their social status. These results show that social isolation results from a combination of intrinsic and environmental factors. From an evolutionary perspective, these findings suggest that social isolation could be adaptive in some contexts and partly maintained by selection.

## Introduction

In gregarious animals, being socially integrated has been linked to reproductive success, health, and longevity^1–4^. Social connections can determine an individual’s access to resources and information, as well as their ability to avoid predators^5,6^. It is perhaps surprising that research from a range of group-living species has shown not all individuals are as integrated in their social environments as all others; studies of humans^7–9^ and other animals^1,10–12^ have documented individuals that are, at least in relation to other members of their population, socially isolated. Isolated individuals may be pushed to the periphery of their social networks by extrinsic factors, such as competitive exclusion. However, social isolation could also be a viable alternative strategy whose benefits arise in contexts, such as elevated risk of communicable disease^13,14^, that are common or severe enough to drive selection but not common enough to be detected by most (relatively short-term) studies relating sociality to fitness. The first critical step toward understanding the evolutionary and neurobiological drivers underlying social isolation is to understand the characteristics of isolated individuals. But we currently know very little about the factors associated with social isolation. For example, whether individuals are consistently isolated over time, whether social isolation is driven by environmental or other factors, or whether isolated animals have features in common with one another, are all currently unclear.

Consistent expression of social isolation within individuals over time, i.e. repeatability, can reveal information about the plasticity of social behaviour and can set the upper limit of its heritable basis^15,16^. This information can, in turn, inform studies of the selective pressures acting on social isolation and of the genomic architecture underlying it. A person’s degree of introversion^17,18^ and feelings of loneliness^19^ may be relatively consistent but whether this consistency translates to being consistently less socially connected is unclear. In nonhuman animals, measures of social integration have been shown to be repeatable in naturalistic settings (e.g. guppies, *Poecilia reticulata* ^20^; sharks, *Scyliorhinus canicula* ^21^) and following experimental perturbations (e.g. beetles, *Bolitotherus cornutus* ^22^; guppies^23^; great tits, *Parus major* ^24^). But barring a few exceptions (kangaroos, *Macropus giganteus* ^25^; great tits^10^), these results tend to be based on two or fewer years of data. Information regarding the consistency of social isolation across longer portions of the lifespan are needed, especially in long-lived species, in order to reveal how social isolation may impact lifetime biological success.

Factors that contribute to social isolation may include the genetic features that drive intra-individual repeatability as well as aspects of the social environment^15^. The social environment experienced early in life may be especially important as it can trigger developmental and epigenetic trajectories that impact phenotypes in later years^26,27^. The role of the environment on phenotypic differences is typically determined by comparing individuals that have had similar experiences^15,16,28^. For the early-life social environment, this includes siblings who share a mother, or animals that were born in the same physical or social space, e.g. the same nest, burrow, or social group. Few studies have examined the role of maternal identity or of other features of the early social environment on behavioural phenotypes because of limitations imposed by data requirements, i.e. maternal identity is not always known and other aspects of the early social environment can be difficult to retrace^29^.

Fundamental biological attributes, such as age, sex, and social status can also contribute to differences in the extent to which individuals are socially isolated^3,30–32^. Kin structure and kin-based dynamics are additionally likely to be important; Members of the non-dispersing sex tend to preferentially interact with kin^33^ and animals that do not have relatives present in their current environment may be more isolated. A lack of familiarity with others may also drive social isolation^34^. Familiarity of the group into which members of the dispersing sex immigrate should increase with time, thus social isolation may be expected to decrease the longer individuals live in a group. Yet it is uncommon in non-captive situations to have data on the amount of time subjects have spent together, and thus the role of familiarity in an individual’s degree of social isolation is not often investigated.

Overall, studies that examine repeatability in an individual’s degree of social isolation, along with the contributions to social isolation derived from the environment experienced in early-life and from fundamental attributes are rare due to a lack of repeated sampling and an absence of information regarding family structure or the conditions experienced in early years. Examining these factors in tandem is especially critical as they are likely intersect and interact in a variety of ways^16^ and each must be evaluated in a manner that takes the others into account.

In this study we examine individual differences in social isolation in a gregarious species of nonhuman primate. We use a large dataset on 429 adult rhesus macaques (*Macaca mulatta*) from 6 different social groups living at the well-studied Cayo Santiago field station in Puerto Rico to determine whether degree of social isolation is repeatable within individuals across years and to examine external factors that influence isolation. These data represent 836 macaque years and span up to 6 years for some individuals, which is roughly half of the median adult lifespan at this site^2^. Some measures of sociality have previously been shown to be heritable in this population^1^ but this work was based on only 2 years of data and thus repeatability was not evaluated. The exceptionally deep demographic database associated with this field site allows us to gain rare insights into the relationship between social isolation and the early social environment, including the identity of an individual’s mother and the group in which individuals were born. Access to long-term demographic data also allows us to explore the relationship between social isolation and familiarity with others, as measured by length of tenure in a group, as well as age, sex, dominance rank, and number of close relatives present.

The extent to which individuals are isolated from their social world is multi-faceted, including a subject’s relationships with others as well as their position in the wider emerging pattern of social connections. Measures used to capture social isolation should therefore reflect this complexity. We used five social network-derived measures to quantify differences in social isolation between individuals, including measures that capture dyadic as well as polyadic, or indirect, social connections^35,36^. Ties in our social network were based on rates of grooming since grooming is one of the most common interactions in gregarious primates and is associated with the formation and maintenance of positive relationships^37^. Dyadic measures of social isolation were instrength and outstrength, which represent the rates at which an individual receives and gives interactions, respectively. In this study, instrength is the rate as which an individual is groomed by others, while outstrength is the rate at which an individual grooms others. Indirect network measures used were betweenness, clustering coefficient and eigenvector centrality. Betweenness is the total number of shortest paths in a network graph that pass through an individual to connect others^38^. Individuals with low betweenness tend not to connect disparate parts of the network to one another. Clustering coefficient represents the nature of an individual’s local network by measuring the proportion of an individual’s social partners who are partners with each other^39^. Animals whose partners are not partners with each other have a clustering coefficient of zero. Finally, eigenvector centrality is a measure of prestige and popularity based on the quality of an individual’s social partners^38^. Individuals with low eigenvector centrality groom infrequently and have partners who groom infrequently. We used a mixed-regression statistical framework^15,28,40,41^ to determine repeatability in degree of social isolation within individuals along with the predictive power of our other factors of interest.

## Results

**Figure 1:**
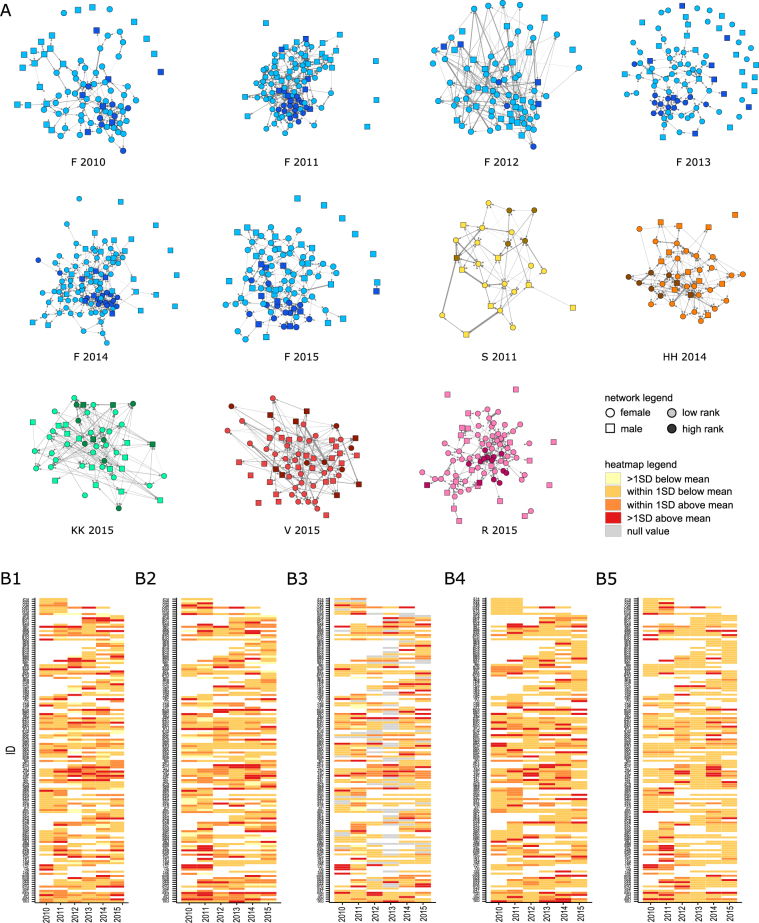
(**A**) Grooming networks constructed using matrices of directional rates of grooming. Social group and year of observation are indicated under each network. Males are squares, females circles. High ranking animals are darker colours. (**B**) Heatmaps of network measures across years for individuals with >2 years of data: (**B1**) instrength, (**B2**) outstrength, (**B3**) clustering coefficient, (**B4**) betweenness, (**B5**) eigenvector centrality. Network measures range from low values (yellow) to high values (red) as shown in legend bars. Blank cells represent years with no data collected for an individual, grey cells in B3 represent animals with one or few social partners and thus null clustering coefficients.

We found that individuals varied in their degree of social isolation. Some animals were not observed to engage in any grooming while others were well connected within their social network (Fig. 1). Network measures are often highly correlated and should not be used as independent terms^35^. We found that no measure was highly collinear with any other, i.e. based on a cut-off of r^2^ > 0.7 in pairwise correlations, and thus retained all five network measures and used each as a dependent variable in our analyses. We used z-scores computed within groups and years for each network measure in order to standardize social isolation relative to the current environment in which an individual was living. Z-scores ranged from −2 to +6 across network measures (Fig. 1), i.e. some animals were two standard deviations below the mean value of a network measure and others were six standard deviations above the mean.

### Repeatability in an individual’s degree of social isolation

**Figure 2:**
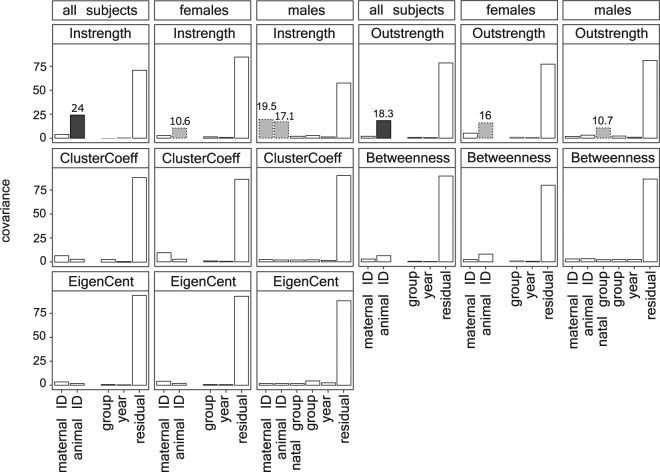
Percentage of variability in network measures of social isolation estimated by variance components: Maternal ID, animal ID, group and year were estimated in models with all subjects. Natal group was additionally examined in models for male subjects. Non-residual values greater than 10% are indicated on plots. Dashed outlines indicate estimates that were greater than 10% but whose lower confidence intervals were too close to zero to be considered robust. Full model results are provided in the Supplementary Materials.

Some measures of social isolation were modestly repeatable (Fig. 1) with >10% of variance explained by individual identity. The amount of grooming given and received to others were 24.0% and 18.3% repeatable, respectively (Fig. 2). These results were found even though features such as maternal effects were accounted for, thus ensuring conservative estimates of individual-level repeatability. No measure of indirect connectedness was repeatable. In addition to our primary model that included all animals, we also generated sex-segregated models that allowed us to explore the factors of biological significance to one sex but not the other. Similar patterns of repeatability emerged when males and females were examined separately, but the credible intervals were too close to zero in all cases to rule out a negligibly small contribution of individual identity (Fig. 2).

### Early-life social environment and degree of social isolation

Maternal effects and the identity of a male’s natal group played negligible roles in explaining the variability in all measures of social isolation examined. Similarities between siblings in their degree of social isolation were generally not explained by the fact that they shared a mother (Fig. 2). Here, it was especially important to explore males and females separately. Adult male rhesus macaques no longer live in their natal group with their mothers and have a dominance rank that is independent from the matriline to which they were born. Investigating males on their own therefore allowed us to determine the association between maternal effects and social isolation independently from the potentially intersecting influences of family presence, network position, and social status that arise in females. When female and male subjects were examined in isolation, we again found that maternal identity was not very informative (Supplementary Materials). The amount of grooming males received from others appeared to demonstrate maternal effects with 19.5% of variability explained, albeit with a lower credible interval too near zero (0.0002) to be considered robust. The identity of the group in which males were born explained below 10% of variability in network position for all measures or had credible intervals nearing zero (Fig. 2).

### Sex, age, dominance rank, and social isolation

**Figure 3:**
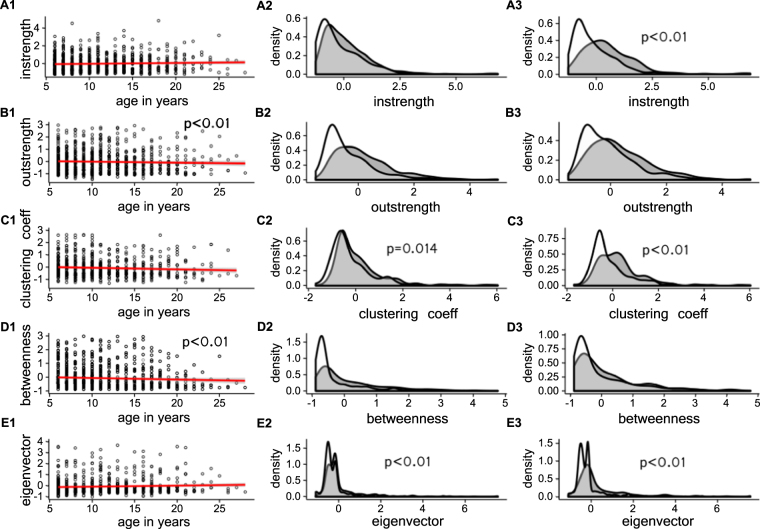
Associations between measures of social isolation and (**A1**–**E1**) age; (**A2**–**E2**) sex, where males are white, females grey; and (**A3**–**E3**) dominance rank, where lower ranking animals are white, high ranking animals grey. Statistically significant relationships are indicated by p-values reported on plots.

Males tended to be more isolated compared to females, especially with respect to indirect connections (Fig. 3) (instrength, estimate = 0.32, p = 0.06; outstrength, estimate = −0.21, p = 0.180; clustering coefficient, estimate = −0.24, p = 0.014; betweenness, estimate = 0.02, p = 0.792; eigenvector, estimate = −0.43, p < 0.001). Age had a weak but significant relationship with some network measures (Fig. 3). Older animals received similar amounts of grooming compared to their younger counterparts (estimate = 0.001, p = 0.888) but spent less time grooming others (estimate = −0.02, p = 0.008) and had lower betweenness (estimate = −0.02, p = 0.004). These results were similar when we considered the sexes separately (Supplementary Materials).

As expected, we found that lower ranking animals were generally more isolated compared to high ranking animals (Fig. 3): they received less grooming (estimate = −0.390, p < 0.001), and had lower eigenvector centrality (estimate = −0.43, p < 0.001) and clustering coefficients (estimate = −0.29, p < 0.001). We found a similar pattern when we examined the sexes in isolation. Low ranking females gave and received less grooming and had lower levels of clustering in their grooming relationships (Supplementary Materials). Lower ranking males were significantly more isolated compared to higher ranking males for almost all measures (Supplementary Materials).

### Family presence, familiarity with groupmates, and social isolation

**Figure 4:**
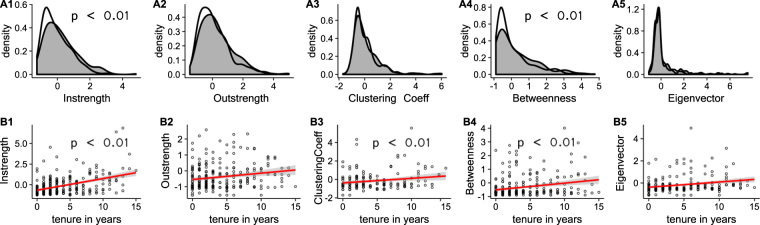
Associations between measures of social isolation and (**A**) presence of adult female relatives for female subjects, (**B**) tenure in current group for male subjects. Number of adult female relatives was reduced to binary presence/absence data for the purposes of visualisation with one or more adult female relatives present represented by grey plots, and no relatives present represented by white plots. Male tenure plots show tenure in years. Statistically significant relationships are indicated by p-values on plots. Full model results can be found in Supplementary Materials.

Females with fewer close adult female relatives received less grooming (estimate = 0.31, p < 0.001) and had lower betweenness (estimate = 0.18, p = 0.018) (Fig. 4). Group tenure predicted males’ network position (Fig. 4); Males who spent a greater number of years in a group received more grooming (estimate = 0.12, p < 0.001), had higher betweenness (estimate = 0.04, p < 0.001) and more clustered relationships (estimate = 0.1, p < 0.001). These results are derived from a model that includes dominance rank and are thus independent from social status. Interestingly, males with longer tenure did not spend more time giving grooming to others (estimate = 0.01, p = 0.560), suggesting they may benefit from their time in the group without investing in return.

### Relationships amongst network measures of social isolation

**Figure 5:**
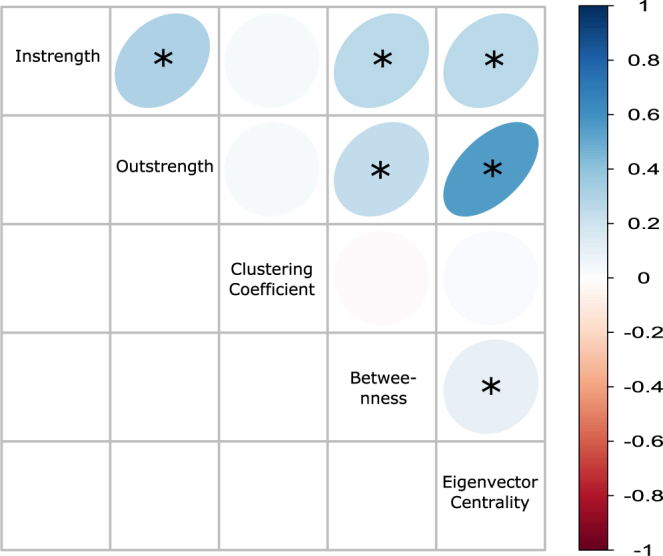
Relationships between measures of social isolation. Red ellipses indicate negative relationships, blue are positive relationships. Darker colours represent stronger relationships, as indicated in the legend on the right. Stars denote statistical significance.

Beyond the importance of establishing their statistical independence, understanding how dyadic and polyadic network measures relate to one another is a key step toward a greater understanding of the importance, if any, of polyadic connections in nonhuman societies^35^. We therefore also examined whether an individual’s degree of social isolation was related across network measures using linear mixed effect models, including animal identity nested within year of data collection as random effect, and age, sex, and dominance rank as fixed effects. We found that the majority of relationships amongst network measures of social isolation had standardised regression coefficients under 0.3, with no coefficients greater than one (Supplementary Materials). In other words, changes of one standard deviation in all measures resulted in changes less than one standard deviation in all other measures (Fig. 5). Indeed, some measures of social isolation were not significant predictors of other measures. For example, the extent to which an individual’s grooming relationships were clustered was not a significant predictor of any other measure (Fig. 5). In other words, animals that were socially isolated with respect to some network measures were nevertheless well integrated according to other measures.

Nonetheless, there were many cases where social isolation according to one measure significantly predicted social isolation according to a different measure. For instance, we found that animals who did not give much grooming also did not received much grooming (Fig. 5). Animals who did not give or receive much grooming were also poorly connected in terms of eigenvector centrality and betweenness (Fig. 5). Because our analyses accounted for other factors, such as dominance rank, these results suggest there may be some common factors that determine social isolation within individuals, regardless of the way isolation is characterised.

## Discussion

There is mounting evidence across a range of species for a positive relationship between social integration and biological benefits, including health, reproductive output and survival^42^. If being socially integrated is beneficial, we must ask why not all individuals are integrated to the same extent. The critical first step toward understanding why individual differences in social isolation exist is to document the characteristics associated with social isolation.

### Are individuals consistently socially isolated?

We found that some social network-based measures of social isolation are moderately repeatable in adult rhesus macaques. Taking common environmental effects and other factors, such as dominance rank, into account, we found that the rates at which individuals groomed others and received grooming from others were moderately repeatable across years. The latter result is important because individuals may be able to exert some control over the interactions they direct outwardly, but are presumed to have less control over the interactions they receive from others^1,7,43^. Consistency in inwardly directed grooming rates across years may therefore suggest individuals are limited in their scope to change their degree of social isolation in some respects.

Our repeatability results are consistent with those from studies of other species. Shoaling tendencies in guppies are repeatable (R = 0.23)^20^, as is the amount of time juvenile sharks spend with others (R = 0.46)^21^ and the number of nearest neighbours in wild kangaroos (R = 0.60)^25^. We note that our repeatability estimates are slightly smaller than those from some previously published reports. This could be the result of the longer-term nature of our data. Many previous studies have focused on 2 or fewer years of data. With the addition of a greater number of years, comes the introduction of greater amounts of variance in the physical and social environments, including the births, deaths, immigrations and emigrations of groupmates. With these changes, it may be reasonable to expect lower repeatability estimates. However, it is also possible that rhesus macaques are simply less resilient to social change^24,44^ and longer expanses of data would continue to reveal higher repeatability estimates in other species. In either case, it is important, particularly in a long-lived species, to examine repeatability across substantial periods of the lifespan. Our data spanned up to half of the median adult lifespan for macaques at this site - 12 years^2^ and thus provide some indication of the scale of the influence social isolation may have across the life course of these animals.

Repeatability takes into account between individual variance that is genetic in both the non-additive (i.e. permanent environmental effects) and additive sense (i.e. heritability)^15,16^. Repeatability estimates can therefore provide (partial) information on the extent to which phenotypes are heritable. Our results align to some extent with the small number of studies that have estimated the heritable basis of social isolation^7,43^. This includes our previous work in the same study population^1^. In that study, we estimated heritability but did not extract repeatability estimates because our data were limited to 2 years. We estimated heritability to be greater than zero for the amount of grooming given, which echoes the repeatability estimate of 18.3% in the current study. In contrast, our previous work estimated heritability of the amount of grooming received to be zero, but in the current study we found a moderate repeatability estimate. One possible explanation for this discrepancy could be that the signal for repeatability we uncovered in the current study is made up entirely of non-additive genetic variance^16^, and thus heritability of this measure is indeed zero as previously reported.

Intriguingly, we found no measure of indirect connectedness to be repeatable. These results contrast the substantial estimates of heritability for two of these measures in our previous work^1^ (betweenness: h^2^ = 0.84; eigenvector centrality h^2^ = 0.36). One possible explanation for this difference lies in differences in the structures of the models in the two studies. In the current study, we modelled maternal effects fully and restricted the effects of natal group to our analysis that excludes females, for which natal group and current group are almost always identical. In contrast, the smaller sample size in our previous work^1^ made it necessary to run only a single model with both sexes combined and therefore to lump all early environmental effects together into a single covariate termed “household effects”. A fuller representation of these variables, along with the greater sample size, could result in more restricted estimates of genetically-related variance^15,16^. However, it is also important to note that variance components are estimated in proportion to the other sources of variability in a quantitative genetic framework. Heritability estimates therefore fluctuate depending on the importance of the environment at a given point in time^16^. The group from which our repeated data are derived experienced a disruptive fission event in 2012, during which some animals were fatally injured^45^. Prior to this period of social upheaval, and in the dataset on which the results from^1^ are based, environmental factors might have been less important in determining indirect connections, thus resulting in a stronger genetic signal. Whereas in the larger, more varied, sample beyond 2012 that is included in the current study, the importance of genetic effects may have been reduced in relation to greater importance taken on by the social environment. Follow-up studies where changes in heritability estimates are evaluated in relation to changes in social context will be a valuable follow up to this work.

### Common environmental effects do not influence differences in social isolation

It was important to examine early-life environmental effects in males and females separately. Because of male-biased dispersal and inter-generational overlap, female rhesus macaques often live with both their mother and their female siblings^33^. As a result, similarities between sisters may be because they shared a common maternal environment early in life, but also may be influenced by the presence of their mother and/or sisters in real time. Males, on the other hand, do not typically live with their family members. Moreover, female rhesus macaques typically acquire the dominance rank directly below their mothers and maintain similar ranks throughout their lives. The dominance rank of males, on the other hand, changes across the lifespan and is more independent from their mother’s rank. Examining maternal effects in males separately from females therefore allowed us to determine the association between maternal effects and social isolation independent from potentially intersecting influences of family presence, network position, and dominance rank.

The early social environment for male rhesus macaques does not, at least measured at this level of coarseness, appear to influence their degree of social integration in adulthood. We found no evidence that the group in which a male was born influences his degree of social isolation later in life. We also found little evidence for maternal effects, defined in this instance as similarities shared between siblings due to the fact that they shared a common environment by sharing a mother. Siblings are not, therefore, similar in their degree of social isolation due to factors associated with having the same mother. This finding is in stark opposition to the recent suggestion that social phenotypes can be understood as a consequence of social inheritance alone^46^ and highlights the importance of allowing both genetic and maternal effects to vary in models of social structure.

### Socially isolated individuals have some attributes in common

We found relationships between social isolation, dominance rank and sex, that would be expected based on previous reports^1,3,32^. Age was negatively related to some measures of social isolation, suggesting that older individuals withdraw from their social worlds, perhaps as a result of reduced energetic reserves. Yet a decline in social integration with age was not always the case, with older animals continuing to receive grooming at the same rate as younger animals. A decline in certain social interactions, such as spending time grooming others, may be beneficial and could, in fact, reflect enhanced knowledge of the environment with age, a pattern that has been previously demonstrated in other species^2,30,47^.

We also found that females with a greater number of close adult female relatives received grooming at a higher rate. Female rhesus macaques have been previously shown to preferentially associate with their close kin^33^, the probabilistic extension of which is that females with a greater number of kin will be less socially isolated^2^. Our results confirm this assumption and thus validate the use of number of close relatives as a proxy of social integration in female rhesus macaques. Using our extensive pedigree and demographic database, we were also able to show that the number of years a male spends in a group negatively correlates with his degree of social isolation, independent from his current dominance rank or age. This may suggest that as males become more familiar with their group mates (and *vice versa*), it becomes easier for them to navigate the social world.

Finally, while some measures of social isolation were weak predictors of other measures, we found this not always to be the case. In other words, while some individuals may be socially isolated in some respects, they are not necessarily isolated in others. This could suggest some common mechanism, e.g. the same simple behavioural rule^48^, driving within individual differences in social isolation but could also reflect a certain level of flexibility in the options afforded to individuals in terms of integration into their social world.

## Conclusions

An improved understanding of the factors that contribute to individual differences in social isolation is a crucial first step towards better understanding why some individuals are more tightly integrated in their social world compared to others. Our results show that individuals can remain socially isolated for considerable portions of their lifespan and that the extent to which an individual is isolated is related partly to their identity and partly to external factors, such as age and familiarity. An extended interpretation of these results is that there may be certain contexts or scenarios in which social isolation is favourable, resulting in the capacity for it to be maintained by selection. For example, social isolation may be beneficial when the risk of communicable disease is elevated^13,14^, or when competition with conspecifics becomes more intense, resulting in an elevated risk of injury for those that frequently associate with others^49^. Research aimed at more firmly establishing the existence of alternative adaptive social strategies with respect to isolation and integration will be of considerable value and will further inform studies of the genetic, neurobiological, and psychological mechanisms that underpin social traits.

## Methods

### Subjects and demographic data

Study subjects were members of the well-studied Cayo Santiago field station off the south eastern coast of Puerto Rico^50^. Subjects were males and females that were mature adults, i.e. six years old or greater^51^, living in six different social groups. We collected data on 429 adults (277 females, 152 males) between the years of 2010–2015, resulting in 836 macaque years (Table 1). For one social group, we collected data in each of the six years of study, while all other groups represented a single year of data. Animals with one year of data cannot contribute to repeatability estimates, but they allow us to evaluate the contribution of other factors, such as maternal identity and dominance rank, with enhanced power. There were 143 individuals with two or more years of data – of these, 127 were animals who were sampled in group F exclusively, and 16 were males that were sampled in group F for one or more years but were also sampled for one year in a different group (Supplementary Materials).

**Table 1:** Total number of adult rhesus macaque subjects per social group and year.

Group	year						Total
	2010	2011	2012	2013	2014	2015	
F	85	100	82	94	104	96	561
HH					43		43
KK						52	52
R						85	85
S		27					27
V						68	68
	**Total number of macaque-years**						**836**
	**Total number of unique individuals**						**429**

We extracted subject age (range = 6–28 years) from the field site’s demographic database, along with natal group and length of tenure in a group for all males. Demographic data are typically collected up to 5 days a week at the site. Females are the philopatric sex in rhesus macaques, with males typically dispersing away from their natal group at sexual maturity and continuing with subsequent dispersals throughout their lives. Males in this study originated from 8 natal groups. Males were considered to have entered a new group once they had been observed in that group for 30 consecutive days. Age and group tenure were measured in years in order to align with our measures of social isolation, which were based on annually recorded data. We recorded tenure as zero for males who entered a study group in the year prior to data collection. Male tenure ranged from 0–15 years.

The identity of the mother of all subjects and the number of close adult female relatives present in a given year for female subjects (coefficient of relatedness = 0.5, based on the degree of relatedness shown to have the strongest association with survival benefits^2^) were extracted from the site’s pedigree database. Details of genetic parentage assignment at this site can be found elsewhere^1,52^.

We collected behavioural data using 10-min focal animal samples^53^ based on a previously established ethogram^1,54^. We recorded the duration of all grooming bouts along with the identity of all social partners. All animals were habituated to observers and individually recognised to the extent that misattribution of the identity of social partners was unlikely. We balanced the number of focal animal samples across individuals within group and year, as well as within subjects across morning and afternoon observations to avoid biases driven by patterns of interactions that occur throughout the day and across the year^55^. We collected a mean number of hours of observation per monkey of 5.70 (SD = 1.98). Variation in the number of hours of data collected per monkey was considerably less within groups and years, ranging from a mean of 3.54 hrs and SD of 0.17 to a mean of 8.97 hrs and SD of 1.45. Monkeys that entered or left a group part way through the year due to death or emigration/immigration were excluded from analyses.

We determined the dominance rank of all subjects using pairwise win-loss information from agonistic encounters that were recorded during focal animal samples or during ad libitum observations. We calculated dominance rank separately for males and females, and within a single group in a given year. In order to account for variable group sizes, we calculated dominance rank as the percentage of groupmates from a subject’s sex that they outranked. We then classed animals as either high or low ranking based on this scale, with high ranking animals being those that outranked >80% of members of their group/sex and low ranking animals being those that were outranked by ≤79% of members of their group/sex^56^.

### Social networks and data analyses

Pairwise grooming rates were the number of seconds a pair of animals spent grooming relative to the number of hours they were observed. We used weighted network measures as these are more robust compared to binary measures^57^. Network measures were calculated using networks comprised of all adult males and females in all cases. We used within group and year z-scores of network measures because it allowed us to evaluate an individual’s degree of social isolation compared to others in their environment instead of evaluating concrete levels of social isolation, which come from an unknown distribution and are more difficult to interpret^16^. This standardization also reduces the likelihood that an animal from a very large or gregarious will appear more socially integrated than animal from a smaller or less social group due to purely demographic processes and mathematical probabilities^54^. Z-scores greater than 1 indicated individuals with network scores 1 standard deviation above the mean in their current group in a given year, while z-scores less than −1 were 1 standard deviation below the mean (Fig. 1).

We estimated repeatability, the impact of maternal and natal group effects, along with the associations between individual attributes on an individual’s degree of social isolation using linear mixed effects models with variance components^40,41,58,59^. For each network measure, we ran 3 separate models: Model 1 included all animals, while models 2 and 3 included only females and only males, respectively (see Supplementary Materials for model specifications). In the model that included all subjects, we modelled maternal identity and individual identity as random effects to estimate maternal effects and individual repeatability, respectively. We also modelled current group and year of data collection as random effects, although we expected neither would explain much variance because our z-score standardization reduced variability driven by these factors. In each case, estimates of the amount of phenotypic variability explained by each term were determined by dividing the variance explained by a term by the variance explained by a combination of all other terms, including error variance^16^. For instance, we estimated repeatability as among-individual variance divided by the sum of within and among individual variance, the variance associated with all other terms in the model, and the error variance. In quantitative genetic terms, repeatability estimated in this manner represents both additive genetic effects, i.e. heritability, as well as non-additive genetic effects, i.e. permanent environmental effects^15,16,28^.

We modelled age, sex, and dominance rank, as fixed effects in the main model. The female-only model included age, dominance rank and number of close adult female relatives as fixed effects, along with individual identity, maternal identity, current group, and year of data collection as random effects. The male-only model included age, dominance rank, and length of tenure in current group as fixed effects, along with individual identity, maternal identity, group and year of data collection, and the identity of the subject’s natal group as random effects. We were not only interested in the associations between these factors and social isolation, but it was also important to take them into account because they can result in what appears to be consistency within individuals^60^ and can thus influence the amount of variation ascribed to individual-level repeatability.

The mixed effect nature of our models allowed us to account for repeated measures of individuals across years as well as estimate repeatability within individuals. We fit these models using Markov chain Monte Carlo (MCMC) routines which, unlike traditional frequentist statistics, cope with the lack of independence between datapoints in social network studies^31,61^. The probability of a value occurring is not tightly tied to its observed frequency in an MCMC framework. When dealing with non-independent data, the distribution of sampled values is therefore broader and more realistic than those generated by frequentist methods, and analyses are less prone to type 1 errors. We examined the interaction terms between all fixed effects but they were only retained in our final models if significant. We report posterior means and credible intervals for all variance components, and estimates with MCMC p-values for all fixed effects.

### Ethical Statement

This research complied with protocols approved by the Institutional Animal Care and Use Committee of the University of Puerto Rico (protocol no. A6850108) and by the University of Exeter School of Psychology’s Ethics Committee.

### Data availability

Data are provided in as a supplementary. csv file.

## Electronic supplementary material

Supplementary Materials
