# Supplementary material for: Persistent social isolation reflects identity and social context but not maternal effects or early environment
Source: Sci Rep. 2017 Dec 19;7:17791. doi: 10.1038/s41598-017-18104-4 (PMC5736592; doi:10.1038/s41598-017-18104-4)
Supplement: Supplementary file 1 — Supplementary Materials [file 41598_2017_18104_MOESM1_ESM.doc]

Supplementary Material

## **Persistent social isolation reflects identity and social context but not maternal effects or early environment**

Brent L.J.N. ^1*^, Ruiz-Lambides A^2^, Platt M.L. ^3^

^1^ School of Psychology, Centre for Research in Animal Behaviour, University of Exeter,

Exeter, U.K.

^2^ Caribbean Primate Research Center, University of Puerto Rico, San Juan, Puerto Rico

^3^ Departments of Neuroscience, Psychology, and Marketing, University of Pennsylvania, Philadelphia, PA, U.S.A.

*Correspondence: L. J. N. Brent, Center for Research in Animal Behaviour, University of Exeter, Perry Road. Exeter EX4 4QG, U.K.

Supplementary Material

Brent et al., Persistent social isolation reflects identity and social context but not maternal effects or early environment

Supplementary Figure S1: Grooming networks across groups and years constructed using matrices of directional rates of grooming. Animal ID appears on node. Males are squares, females circles. Colours are number of years an animal was sampled. N = 16 males were sampled in more than one group.


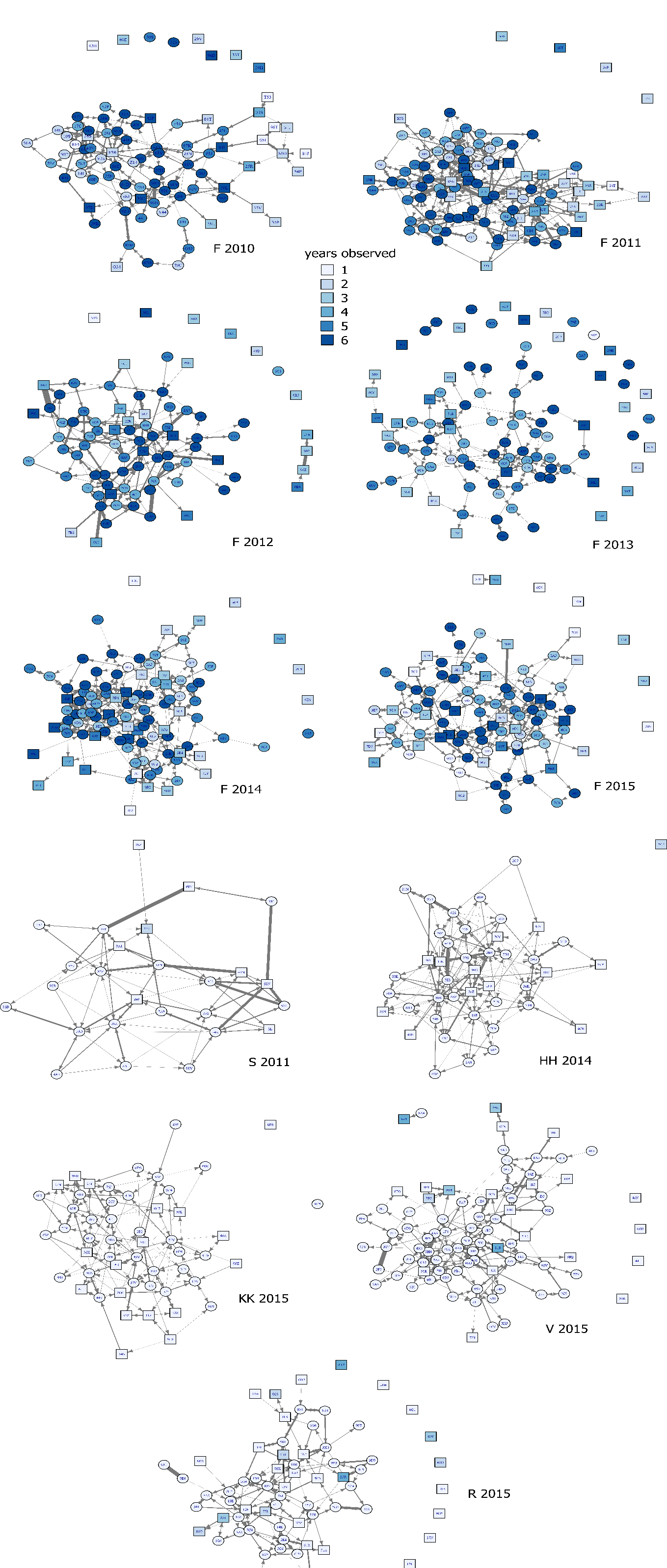


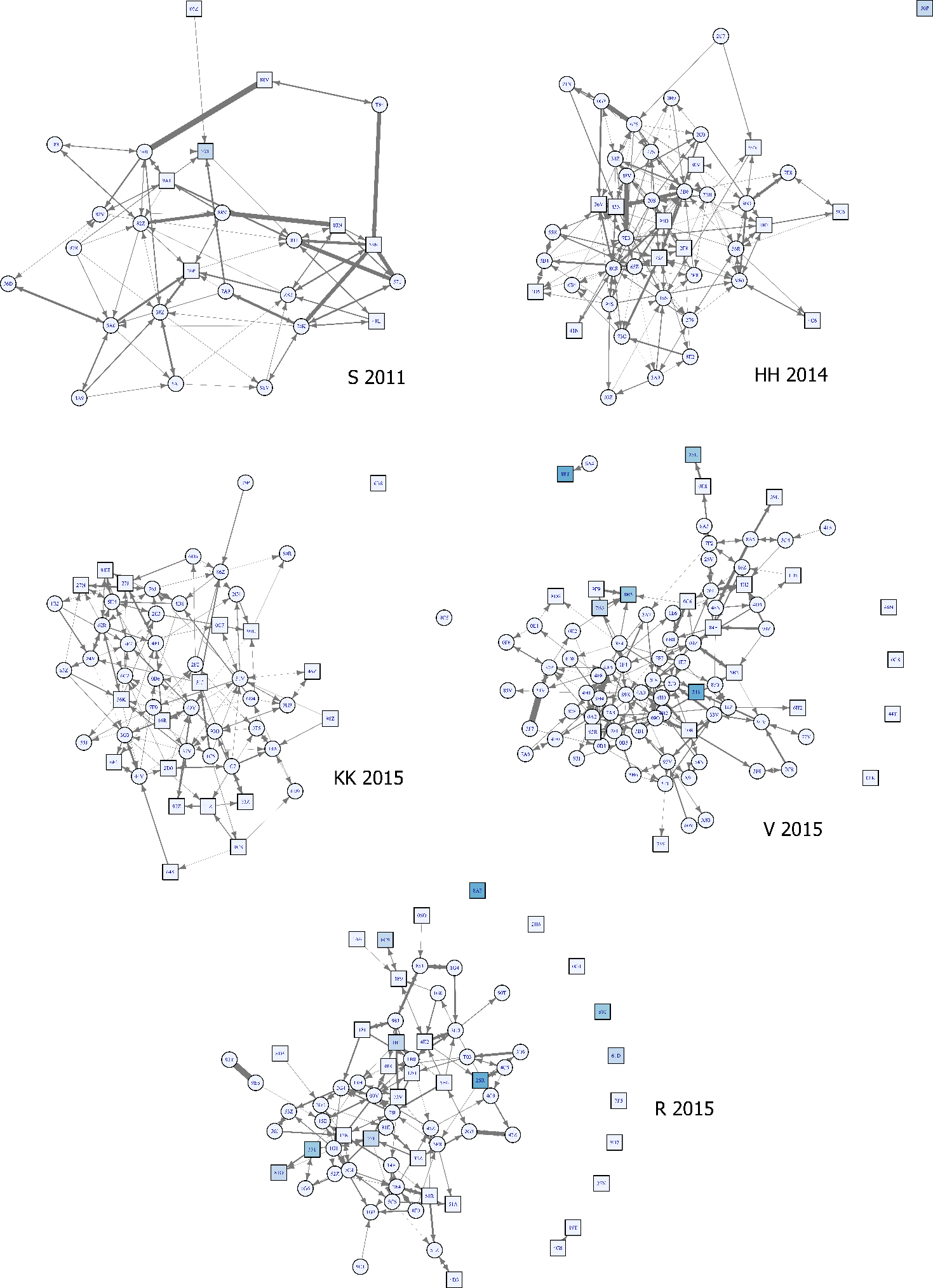


Table S1: Specifications of statistical models. Models were run with each of the five network measures as the dependent variable. Network measures were calculated using networks comprised of all adult males and females in all cases.

|  | Covariates | | | | | | Fixed Effects | | | | | |
| --- | --- | --- | --- | --- | --- | --- | --- | --- | --- | --- | --- | --- |
| **Subjects included in model** | **Maternal ID** | **Animal ID** | **Current group** | **Current year** | **Natal group** |  | | **sex** | **age** | **Dominance**  **rank** | **Tenure in group** | **# AF family members in group** |
| All subjects | x | x | x | x |  |  |  | x | x | x |  |  |
| Females only | x | x | x | x |  |  |  |  | x | x |  | x |
| Males only | x | x | x | x | x |  |  |  | x | x | x |  |

Table S2: Covariance effects for network measures in model including all subjects. Values are posterior means with credible intervals in ().

| **Network metric** | **Maternal ID** | **Repeatability**  **(animal ID)** | **Current Group** | **Year** | **Residual variance** |
| --- | --- | --- | --- | --- | --- |
| Instrength | 0.039 (0.0003, 0.124) | 0.240 (0.111, 0.374) | 0.0007 (0.0002, 0.024) | 0.004 (0.0002, 0.014) | 0.709 (0.611, 0.813) |
| Outstrength | 0.020 (0.0005, 0.073) | 0.183 (0.093, 0.286) | 0.007 (0.0002, 0.023) | 0.005 (0.0003, 0.016) | 0.785 (0.693, 0.872) |
| Clustering coeff | 0.069 (0.0002, 0.145) | 0.029 (0.0002, 0.105) | 0.027 (0.0002, 0.105) | 0.005 (0.0002, 0.015) | 0.891 (0.812, 0.970) |
| Betweenness | 0.030 (0.0004, 0.084) | 0.064 (0.0008, 0.141) | 0.006 (0.0003, 0.024) | 0.004 (0.0002, 0.015) | 0.896 (0.818, 0.966) |
| Eigenvector | 0.034 (0.0003, 0.093) | 0.017 (0.0002, 0.057) | 0.007 (0.0002, 0.022) | 0.004 (0.0002, 0.014) | 0.937 (0.871, 0.991) |

Table S3: Relationships between network measures and animal attributes in a model including both male and female subjects. Values are regression estimates with MCMC p-values.

| **Network metric** | **age** | **sex** | **rank** | **sex*rank** | **age*sex** |
| --- | --- | --- | --- | --- | --- |
| Instrength | 0.001, p = 0.888 | 0.317, p = 0.056 | -0.390, p < 0.0001 | -0.624, p < 0.001 | N.S. |
| Outstrength | -0.023, p = 0.008 | -0.213, p = 0.180 | -0.199, p = 0.070 | -0.511, p = 0.006 | N.S. |
| Clustering coeff | -0.011, p = 0.234 | -0.236, p = 0.014 | -0.292, p < 0.001 | N.S. | N.S. |
| Betweenness | -0.022, p = 0.004 | -0.018, p = 0.940 | 0.024, p = 0.792 | -0.574, p < 0.001 | N.S. |
| Eigenvector | 0.0003, p = 0.972 | -0.331, p < 0.001 | -0.434, p < 0.001 | NS | N.S. |

Table S4: Covariance effects for network measures in model including female subjects only. Values are posterior means with credible intervals in ().

| **Network metric** | **Maternal ID** | **Repeatability (animal ID)** | **Current Group** | **Year** | **Residual variance** |
| --- | --- | --- | --- | --- | --- |
| Instrength | 0.026 (0.0004, 0.092) | 0.106 (0.0001, 0.209) | 0.014 (0.0003, 0.057) | 0.008 (0.0002, 0.027) | 0.846 (0.733, 0.945) |
| Outstrength | 0.052 (0.0004, 0.184) | 0.160 (0.0006, 0.283) | 0.010 (0.0002, 0.037) | 0.005 (0.0002, 0.018) | 0.772 (0.666, 0.881) |
| Clustering coeff | 0.097 (0.0008, 0.192) | 0.028 (0.0002, 0.115) | 0.010 (0.0003, 0.034) | 0.006 (0.0001, 0.018) | 0.861 (0.756, 0.964) |
| Betweenness | 0.023 (0.0002, 0.081) | 0.081 (0.0009, 0.176) | 0.010 (0.0002, 0.033) | 0.006 (0.0002, 0.020) | 0.800 (0.782, 0.981) |
| Eigenvector | 0.041 (0.0002, 0.125) | 0.018 (0.001, 0.063) | 0.007 (0.0001, 0.026) | 0.006 (0.002, 0.022) | 0.928 (0.843, 0.992) |

Table S5: Relationships between network measures and attributes of female subjects. Values are regression estimates with MCMC p-values.

| **Network metric** | **age** | **rank** | **No.**  **AFrelatives** | **age*rank** | **rank* AFrelatives** | **age* AFrelatives** |
| --- | --- | --- | --- | --- | --- | --- |
| Instrength | 0.002, p = 0.818 | -0.450, p <0.001 | 0.314, p < 0.001 | N.S. | N.S. | N.S. |
| Outstrength | -0.024 , p = 0.014 | -0.370, p = 0.006 | -0.209, p = 0.198 | N.S. | 0.427, p = 0.014 | N.S. |
| Clustering coeff | -0.003, p = 0.714 | -0.338, p = 0.006 | -0.021, p = 0.772 | N.S. | N.S. | N.S. |
| Betweenness | -0.022, p = 0.040 | 0.011, p = 0.954 | 0.179, p = 0.018 | N.S. | N.S. | N.S. |
| Eigenvector | -0.001, p = 0.896 | -0.345, p = 0.008 | 0.168, p = 0.022 | N.S. | N.S. | N.S. |

Table S6: Covariance effects for network measures in model including male subjects only. Values are posterior means with credible intervals in ().

| **Network metric** | **Maternal ID** | **Repeatability (animal ID)** | **Current Group** | **Year** | **Natal group** | **Residual variance** |
| --- | --- | --- | --- | --- | --- | --- |
| Instrength | 0.195 (0.0002, 0.434) | 0.171 (0.0001, 0.425) | 0.026 (0.0002, 0.111) | 0.013 (0.0002, 0.049) | 0.020 (0.0002, 0.079) | 0.575 (0.419, 0.745) |
| Outstrength | 0.019 (0.0002, 0.068) | 0.031 (0.0002, 0.110) | 0.022 (0.0001, 0.092) | 0.010 (0.0003, 0.035) | 0.107 (0.001, 0.350) | 0.811 (0.570, 0.978) |
| Clustering coeff | 0.023 (0.0002, 0.100) | 0.020 (0.0003, 0.079) | 0.021 (0.0002, 0.088) | 0.016 (0.0003, 0.064) | 0.020 (0.0002, 0.088) | 0.900 (0.755, 0.994) |
| Betweenness | 0.031 (0.0004, 0.105) | 0.0334 (0.0004, 0.112) | 0.025 (0.0003, 0.111) | 0.024 (0.0002, 0.088) | 0.022 (0.0004, 0.084) | 0.864 (0.719, 0.977) |
| Eigenvector | 0.016 (0.0005, 0.054) | 0.017 (0.0004, 0.055) | 0.044 (0.0005, 0.188) | 0.025 (0.0005, 0.088) | 0.017 (0.0004, 0.060) | 0.881 (0.724, 0.983) |

Table S7: Relationship between network measures and attributes of male subjects. Values are regression estimates with MCMC p-values.

| **Network metric** | **age** | **rank** | **group  tenure** | **age*rank** | **rank*tenure** | **age*tenure** |
| --- | --- | --- | --- | --- | --- | --- |
| Instrength | -0.040, p = 0.068 | -0.598, p < 0.001 | 0.123, p < 0.001 | N.S. | N.S. | N.S. |
| Outstrength | -0.027, p = 0.054 | -0.683, p < 0.001 | 0.010, p = 0.560 | N.S. | N.S. | N.S. |
| Clustering coeff | -0.08, p = 0.006 | 0.077, p = 0.748 | 0.098, p < 0.001 | N.S. | N.S. | N.S. |
| Betweenness | -0.031, p = 0.022 | -0.400, p = 0.010 | 0.038, p = 0.038 | N.S. | N.S. | N.S. |
| Eigenvector | -0.017, p = 0.110 | -0.684, p < 0.001 | 0.006, p = 0.662 | N.S. | N.S. | N.S. |

Table S8: Relationships between grooming network measures of centrality.

**Values are regression estimates with MCMC p-values. Models control for age, sex & dominance rank.**

|  | G.InS | G.OutS | G.CCF | G.Bet | G.Eigen |
| --- | --- | --- | --- | --- | --- |
| Instrength |  | 0.305 (<0.001) | 0.035 (0.356 | 0.266 (<0.001) | 0.262 (<0.001) |
| Outstrength |  |  | 0.036 (0.341) | 0.247 (<0.001) | 0.558 (<0.001) |
| Clustering coeff |  |  |  | -0.020 (0.596) | 0.029 (0.449) |
| Betweenness |  |  |  |  | 0.091 (0.006) |
| Eigenvector |  |  |  |  |  |
